# Characterization of the complete chloroplast genome of *Ephedra sinica* Stapf (Ephedraceae), a traditional Chinese medicine

**DOI:** 10.1080/23802359.2019.1673233

**Published:** 2019-10-01

**Authors:** Peng Li, Jinyang Zhang, Xiaoqing Liang

**Affiliations:** Department of Pharmacy, Xi’an International University, Xi’an, Shaanxi, China

**Keywords:** *Ephedra sinica* Stapf, Ephedraceae, chloroplast genome, Illumina sequencing, phylogenetic analysis

## Abstract

*Ephedra sinica* Stapf is a traditional Chinese medicine of Ephedraceae in China, which contains many chemicals, such as ephedrine and pseudoephedrine, flavonoids, polysaccharides, phenolic compounds, and proanthocyanidins. It shows significant activity in asthma, fever, rheumatoid arthritis, and also promotes diuresis and sweat.

Illumina paired-end reads data was used to assemble the complete chloroplast (cp) genome. The length of circular cp genome is 109,525 bp, including a large single-copy (LSC) region of 59,960 bp, a small single-copy (SSC) region of 8,079 bp, and a pair of inverted repeat (IRs) regions of 20,743 bp. Besides, 11 genes possess a single intron, while the gene *ycf3* has a couple of introns. The GC content of entire Ephedra sinica Stapfcp genome, LSC, SSC and IR regions are 36.7, 34.2, 27.7, and 42.0%, respectively. Based on the concatenated coding sequences of cp PCGs, the phylogenetic analysis showed that *Ephedra sinica* Stapf and *Ephedra foeminea* (KT934791) are closely related with each other within the family Compositae.

*Ephedra sinica* Stapf is a traditional Chinese medicine of Ephedraceae in China (Liang et al. 2018), which contains many chemicals, such as ephedrine and pseudoephedrine (Andraws et al. 2005; Ma et al. 2007), flavonoids (Amakura et al. 2013), polysaccharides (Zhao et al. 2009), phenolic compounds (Cottiglia et al. 2005) and proanthocyanidins (Zang et al. 2013). It shows significant activity in asthma, fever, cough, rheumatoid arthritis (Wang et al. 2016), and also promotes diuresis and sweat (Ma et al. 2018).

A pair of inverted repeats (IRs), separated by a large single-copy region (LSC) and a small single-copy region (SSC), these four parts constitute a conserved structure of the complete cp genome (Wolfe et al. 1992; Lee et al. 2007). This report will be very important for studying the phylogenetic relationships of *E. sinica* Stapf and Ephedraceae.

The fresh leaves of *E. sinica* Stapf were collected in the Ningxia Forestry Institute (38°28′N, 106°16 106ia Fore, NW China). A voucher specimen (FS190409) was deposited at Pharmacognosy laboratory in Xi’an International University. The modified CTAB method was used to extract the genomic DNA (Doyle and Doyle 1987). We constructed a shotgun library with Illumina HiSeq X Ten Sequencing System (Illumina, San Diego, CA) following the manufacturer’s specification. The program MITObim v1.8 (https://github.com/chrishah/MITObim) was used to assemble cp genome (Hahn et al. 2013) and *E. foeminea* (GenBank: KT934791) as the initial reference. The map of the complete cp genome was generated through the web-based tool OGDRaw v1.2 (http://ogdraw.mpimp-golm.mpg.de/) (Lohse et al. 2013) and the complete cp genome sequence has been submitted to GenBank (accession number MNI199030).

The complete cp genome is a circular double stranded DNA molecule, with a typical quadripartite structure, including a pair of IRs, an LSC region, and an SSC region. We got 17,104,768 raw paired-end reads and the length distribution in 109,525 bp (GC content accounts for 36.7%), furthermore, the lengths of LSC region, IRs regions, and SSC region distribution are 59,960 bp (GC, 34.2%), 20,743 bp (GC, 42.0%), and 8079 bp (GC, 27.7%), respectively.

The sequencing result encodes 101 complete genes, containing 67 protein-coding genes, 30 transfer RNA genes, and 4 ribosomal RNA genes. In addition, 4 tRNA genes (*trnA-UGC*, *trnI-GAU*, *trnK-UUU* and *trnL-UAA*) harbor a single intron. *AtpF*, *petB*, *petD*, *rpl2*, *rpl16*, *rpoC1*, *rps12*, these 7 PCG genes possess a single intron, 53 PCG genes no intron, *ycf3* harbor two introns.

Based on the concatenated coding sequences of 10 cp PCGs for 8 plastid genomes from published species of Gnetidae, and two other species, *Cupressus jiangeensis* (MG596347) and *Juniperus cedrus* (KT378453), were included as outgroup, we constructed a neighbour-joining (NJ) phylogenetic tree (Figure 1) using MEGA7 with 1000 bootstrap replicates (Kumar et al. 2016) (http://www.megasoftware.net/) to further study the phylogenetic position of *E. sinica* Stapf. From the NJ phylogenetic tree analysis, we find that *E. sinica* and *E. foeminea* (KT934791) are closely related to each other within the family Ephedraceae.

**Figure 1.**
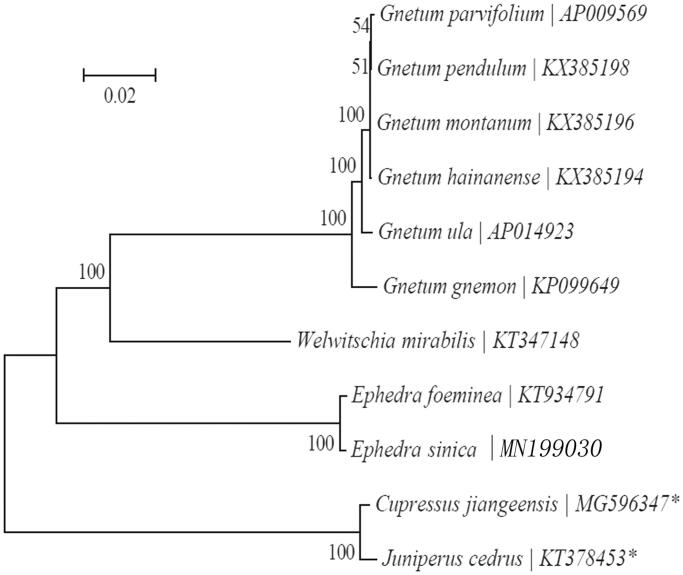
Maximum-likelihood (ML) tree of *Ephedra sinica* and its related relatives based on the complete cp genome sequences. *represents the outgroup of Gnetidae.
